# Association of sleep quality and nap duration with cognitive frailty among older adults living in nursing homes

**DOI:** 10.3389/fpubh.2022.963105

**Published:** 2022-08-25

**Authors:** Siyue Liu, Zhao Hu, Yicong Guo, Feixiang Zhou, Shaojie Li, Huilan Xu

**Affiliations:** Department of Social Medicine and Health Management, Xiangya School of Public Health, Central South University, Changsha, China

**Keywords:** cognitive frailty, sleep quality, nap duration, nursing homes, older adults

## Abstract

**Background:**

Sleep status, including sleep quality and nap duration, may be associated with frailty and cognitive impairment in older adults. Older adults living in nursing homes may be more prone to physical and cognitive frailties. This study aimed to investigate the association between sleep quality and nap duration, and cognitive frailty among older adults living in nursing homes.

**Methods:**

This study included 1,206 older adults aged ≥ 60 years from nursing homes in Hunan province, China. A simple frailty questionnaire (FRAIL scale) was used and Mini-Mental State Examination was conducted to assess physical frailty and cognitive impairment, respectively, to confirm cognitive frailty. The Pittsburgh Sleep Quality Index was used to assess the sleep quality. Nap duration was classified as follows: no, short (≤30 min), and long (>30 min) napping. Multinomial logistic regression was conducted to estimate the odds ratio (OR) and 95% confidence interval (CI).

**Results:**

The prevalence of cognitive frailty among the older adults in nursing homes was 17.5%. Approximately 60.9% of the older adults had a poor sleep quality. Among the 1,206 participants, 43.9% did not take naps, 29.1% had short naps, and 26.9% had long naps. After adjusting for all covariates, poor sleep quality (OR 2.53; 95% CI 1.78–3.59; *P* < 0.001) and long nap duration (OR 1.77; 95% CI 1.19–2.64; *P* = 0.003) were associated with higher odds of cognitive frailty, but short nap duration (OR 0.60; 95% CI 0.40–0.89; *P* = 0.012) was associated with low prevalence of cognitive frailty.

**Conclusion:**

Poor sleep quality and long nap duration are significantly associated with high risk of cognitive frailty among the older adults in nursing homes. Short nap duration was associated with low prevalence of cognitive frailty. However, these associations require further validation in older adults.

**Clinical trial registration:**

https://osf.io/57hv8.

## Introduction

The process of global aging is accelerating, and the global population of older adults is increasing with the development of society and the improvement of medical technology. The family pension system is the most traditional pension system in China, but the family pension function cannot take on new functions because of changes in Chinese family patterns (1). In addition, older adults with lower self-care abilities tend to choose nursing homes that can provide professional care because of poorer health and increased life expectancy. Nursing homes in China rely on social resources to provide institutional care for older adults as a social welfare system (2). Therefore, an increasing number of older adults will choose to live in nursing homes.

As a subtype of frailty (3), cognitive frailty has recently become a new research direction in the field of modern geriatric medicine. Cognitive frailty was first proposed by an international consensus group in 2013, and it refers to a heterogeneous clinical manifestation characterized by simultaneous presence of both physical frailty and cognitive impairment, excluding Alzheimer's disease or other dementia diseases (4). On this basis, Ruan et al. further refined the concept of cognitive frailty in 2015, and cognitive impairment in their definition was caused by physical pre-frailty and physical frailty (5). Many studies have found that cognitive frailty is common among older adults, although studies in this field are still in the preliminary stage. Generally, the prevalence of cognitive frailty in community-dwelling older adults is 9% (6), and that in Chinese nursing homes is 27.1% (7). In addition, cognitive frailty is associated with high risk of a range of adverse outcomes such as malnutrition (8), negative emotional problems (9), disability (10), and death (11).

It has become an academic consensus that sleep status is related to the health of older adults, particularly sleep quality and nap duration. A previous study showed that in nursing home, the prevalence of poor sleep quality among older residents is 60.3% (12). In Chinese nursing homes, the prevalence of poor sleep quality among older adults is 67.3% (13). Sleep problems have a significant impact on the functional status and quality of life of older adults (14) and even increase the risk of mortality (15). Naps are also an important component of individual sleep status. A review of the literature showed that the proportion of older adults taking naps ranges from 20 to 60% worldwide, and that it has exceeded 55% of older adults in China (16). In addition, long nap duration, from numerous studies, was associated with many adverse outcomes in older adults, including depressive symptoms (17) and diabetes (18).

Current studies have explored factors related to cognitive frailty in order to provide a theoretical basis for prevention. It has been shown that various factors are associated with cognitive frailty, such as physical activity (19) and nutrition (20). Moreover, many studies have shown that both physical frailty and cognitive impairment are associated with sleep status, including sleep quality (21, 22) and nap duration (23, 24). There are still insufficient studies related to the association between sleep status and cognitive frailty, except for a study on the association of nighttime sleep duration with cognitive frailty among community-dwelling older adults in 2021 (25). Few studies have systematically investigated the association between sleep quality and nap duration, and cognitive frailty among older adults in nursing homes.

Sleep status plays an important role in health as a part of people's daily lives. Early screening and intervention have the positive significance for the health of older adults, considering that sleep status can be improved. Simultaneously, older adults in nursing homes are a potential group of individuals with cognitive frailty, because they are in the context of population aging. And cognitive frailty is reversible (26). Therefore, this study aimed to target older adults in nursing homes in Hunan province and investigate the association between sleep quality and nap duration, and cognitive frailty among older adults. This study provides evidence for promoting the physical and cognitive status of older adults in nursing homes in the field of sleep health, including sleep quality and nap duration.

## Methods

### Participants

In this study, a multistage sampling method was used to select a representative sample of older adults in nursing homes in Hunan province. First, we selected cities in western, northern, southern, and central Hunan (i.e., divided by region): Huaihua, Shaoyang, Changde, and Xiangtan cities. Subsequently, half of the counties/districts in each city were randomly selected. This study included all nursing homes in each selected county/district, for a total of 15 nursing homes. Finally, all older adults eligible for this study were recruited from selected nursing homes. Older adults in the selected nursing homes were included in our study if they met the following inclusion criteria: (1) age 60 years or older; (2) duration of entrance into nursing homes of ≥3 months; and (3) communicated and voluntarily cooperated to participate in this study. However, older adults were excluded if they (1) were unconscious or comatose, (2) suffered from severe illness (e.g., heart failure in stage IV, tumor with multiple metastases, chronic renal failure in the uremic stage, and other serious organic diseases), (3) had audiovisual impairment and language communication difficulties, and (4) had Alzheimer's disease or other types of dementia.

This was a cross-sectional study, and the protocol was registered on the Open Science Framework (Registration doi: https://doi.org/10.17605/OSF.IO/57HV8).

From July 2021 to December 2021, 1,565 older adults were recruited for this study with the support of the Civil Affairs Bureau. Among them, 29 participants lived for <3 months, and 322 participants diagnosed with Alzheimer's disease, and 8 participants with missing data. Finally, we included a total of 1,206 participants.

### Measurements

#### Physical frailty assessment

We used a simple frailty questionnaire (FRAIL scale) to assess the physical frailty of the participants (27). The scale has 5 items: fatigue, resistance, ambulation, illnesses, and loss of weight. Dong et al. validated the Chinese version of the FRAIL scale for older adults in China, and the results showed that it had good sensitivity, specificity, test-retest reliability, and internal consistency (28).

Each item is scored with 0–1 point, and they require a “yes” or “no” answer which one point was given to any positive response. Finally, the scores of all the items were accumulated, and the total score range was 0–5 points. Physical frailty was indicated when the total score on the FRAIL scale was ≥3 points, and physical pre-frailty was indicated when the total score was 1 or 2 points.

#### Cognitive function assessment

Mini-Mental State Examination (MMSE) was conducted to assess cognitive function (29). The scale consists of 30 questions and includes five dimensions: orientation, memory, attention and calculation, recall, and language ability. The Chinese version of MMSE also shows good reliability and validity (30).

Each question was scored 1 or 0 point for correct or incorrect answers/non-responsive, respectively, and the total score was in the range of 0–30 points. According to the education of the participants, this study used MMSE to evaluate cognitive impairment criteria as follows: illiteracy ≤ 17 points, primary school ≤ 20 points, and secondary school and above ≤24 points (31).

#### Definition of cognitive frailty

In our study, cognitive frailty was defined as the presence of both cognitive impairment and physical pre-frailty or physical frailty following Ruan's proposed definition (5), which means it meets both the results of measures: the MMSE score demonstrates cognitive impairment, and the FRAIL scale score is ≥1.

Referring to previous study groups (25, 32), our study divided the included participants into five groups according to physical frailty and cognitive function status: (1) robust group: neither had physical frailty, physical pre-frailty, nor cognitive impairment; (2) physical pre-frailty group: older adults with only physical pre-frailty and without cognitive impairment; (3) physical frailty group: older adults with only physical frailty and no cognitive impairment; (4) cognitive impairment group: older adults who only had cognitive impairment but neither had physical pre-frailty nor physical frailty; (5) cognitive frailty group: older adults with both cognitive impairment and physical pre-frailty or physical frailty.

#### Sleep quality assessment

The Pittsburgh Sleep Quality Index (PSQI) was used in our study to investigate the sleep quality of older adults over the past month (33). The PSQI covers seven components: subjective sleep quality, sleep latency, sleep duration, habitual sleep efficiency, sleep disturbances, use of sleeping medication, and daytime dysfunction. The Chinese version of the PSQI has also been effectively tested in older adults in China (34). The results showed that the PSQI has good reliability and validity, with a Cronbach's α coefficient of 0.68.

The score for each dimension ranged from 0 to 3 points, and the final PSQI total score was accumulated with the scores of each dimension, with a full score of 21 points. In addition, higher PSQI scores indicate poor sleep quality. A PSQI score of >7 indicates poor sleep quality (34).

#### Nap duration assessment

We used the following question: “During the past month, how long did you usually nap?” to collect data on nap duration from the participants. In this study, napping only referred to take a nap after lunch. After reviewing the literature (35), 30 min was found to be a common cut-off time for naps. Thus, the participants were divided into three groups: (1) no napping: the participants did not take naps; (2) short nap duration: nap duration ≤ 30 min; and (3) long nap duration: nap duration > 30 min.

#### Covariates

The covariates included sociodemographic information, lifestyle behaviors, and health-related conditions. Sociodemographic information included age, sex, ethnicity, residence, education, marital status, economic source, income, and number of children. Education was collected by asking the participants about their highest educational level, and divided into illiteracy, primary school, secondary school and above. Marital status was divided into married, widowed, and other. Income was divided into ≤ 2,000 RMB (Ren Min Bi), 2,001–5,000 RMB and ≥ 5,001 RMB. Lifestyle behaviors included smoking history (never/former/current) and drinking history (never/current) over the past month. Health-related conditions included number of chronic diseases (0/1/2/≥3), medicines taken (0/1/≥2), painful areas (0/1/≥2), and depressive symptoms assessed with Geriatric Depression Scale-15 (GDS-15) (36). This indicates that older adults have depressive symptoms when the GDS-15 score is ≥8 points. The study showed that GDS-15 has good reliability and validity for older adults in China, with a Cronbach's α coefficient of 0.79 (37).

### Statistical methods

All the analyses were performed using IBM SPSS version 26.0. Categorical variables were described as n (%), and continuous variables were described as mean ± standard deviation (SD). The characteristics of the participants were presented according to physical frailty and cognitive function status, and differences among the five groups were compared by chi-square test and one-way analysis of variance (ANOVA). Taking cognitive frailty as the dependent variable, multinomial logistic regression was conducted to analyze the association between sleep status and cognitive frailty among older adults in nursing homes. The adjustments for potential confounders included age, sex, ethnicity, residence, education, marital status, economic source, income, number of children, smoking history, drinking history, number of chronic diseases, number of medicines taken, number of painful areas, and depressive symptoms. The odds ratio (OR) and 95% confidence interval (CI) were conducted. *P* < 0.05 was specified to determine the significant effect.

## Results

A total of 1,206 participants, including 597 men and 609 women, were included in our study. The age of the participants was 77.32 ± 8.87 years. In our study, 60.9% (*n* = 735) of the participants with poor sleep quality. A total of 43.9% (*n* = 530) of the older adults did not take naps, and 29.1% (*n* = 351) and 26.9% (*n* = 325) reported short and long nap durations, respectively. In addition, the study found that 17.5% of the participants (*n* = 211) had cognitive frailty, 16.7% (*n* = 202) had cognitive impairment, and 11.8% (*n* = 142) had physical pre-frailty without cognitive impairment. Physical frailty with intact cognitive function accounted for 15.1% (*n* = 182) of the participants. Additional details are presented in Table 1.

**Table 1:** Characteristics of the participants according to physical frailty and cognitive function status.

**Characteristics**	**Overall** **(*n* = 1,206)**	**Robust** **(*n* = 469)**	**Physical pre-frailty** **(*n* = 142)**	**Physical frailty** **(*n* = 182)**	**Cognitive impairment** **(*n* = 202)**	**Cognitive frailty** **(*n* = 211)**	***P*-value**
Age (years)^*^	77.32 ± 8.87	76.11 ± 8.66	78.30 ± 8.21	79.75 ± 8.63	76.43 ± 8.83	78.11 ± 9.49	<0.001^**^
Age (years) (*n*, %)							<0.001
60–69	273 (22.6)	125 (26.7)	26 (18.3)	26 (14.3)	53 (26.2)	43 (20.4)	
70–79	378 (31.3)	163 (34.8)	41 (28.9)	45 (24.7)	66 (32.7)	63 (29.9)	
80–89	473 (39.2)	154 (32.8)	68 (47.9)	98 (53.8)	71 (35.1)	82 (38.9)	
≥90	82 (6.8)	27 (5.8)	7 (4.9)	13 (7.1)	12 (5.9)	23 (10.9)	
Sex (*n*, %)							0.086
Males	597 (49.5)	252 (53.7)	73 (51.4)	78 (42.9)	92 (45.5)	102 (48.3)	
Females	609 (50.5)	217 (46.3)	69 (48.6)	104 (57.1)	110 (54.5)	109 (51.7)	
Ethnicity (*n*, %)							0.632
Han	1,186 (98.3)	460 (98.1)	138 (97.2)	179 (98.4)	200 (99.0)	209 (99.1)	
National minority	20 (1.7)	9 (1.9)	4 (2.8)	3 (1.6)	2 (1.0)	2 (0.9)	
Residence (*n*, %)							0.058
Urban	798 (66.2)	295 (62.9)	93 (65.5)	122 (67.0)	151 (74.8)	137 (64.9)	
Rural	408 (33.8)	174 (37.1)	49 (34.5)	60 (30.0)	51 (25.2)	74 (35.1)	
Education (*n*, %)							<0.001
Illiteracy	110 (9.1)	55 (11.7)	17 (12.0)	18 (9.9)	2 (1.0)	18 (8.5)	
Primary school	647 (53.6)	272 (58.0)	66 (46.5)	80 (44.0)	122 (60.4)	107 (50.7)	
Secondary school and above	449 (37.2)	142 (30.3)	59 (41.5)	84 (46.2)	78 (38.6)	86 (40.8)	
Marital status (*n*, %)							<0.001
Married	507 (42.0)	217 (46.3)	56 (39.4)	42 (23.1)	97 (48.0)	95 (45.0)	
Widowed	575 (47.7)	177 (37.7)	82 (57.7)	132 (72.5)	82 (40.6)	102 (48.3)	
Others	124 (10.3)	75 (16.0)	4 (2.8)	8 (4.4)	23 (11.4)	14 (6.6)	
Economical source (*n*, %)							<0.001
Pensions	836 (69.3)	320 (68.2)	93 (65.5)	111 (61.0)	159 (78.7)	153 (72.5)	
Family supports	321 (26.6)	115 (24.5)	47 (33.1)	69 (37.9)	40 (19.8)	50 (23.7)	
Others	49 (4.1)	34 (7.2)	2 (1.4)	2 (1.1)	3 (1.5)	8 (3.8)	
Income (*n*, %)							0.650
≤ 2,000 RMB	40 (3.3)	12 (2.6)	7 (4.9)	6 (3.3)	9 (4.5)	6 (2.8)	
2,000–5,000 RMB	1,114 (92.4)	442 (94.2)	127 (89.4)	166 (91.2)	183 (90.6)	196 (92.9)	
≥5,001 RMB	52 (4.3)	15 (3.2)	8 (5.6)	10 (5.5)	10 (5.0)	9 (4.3)	
Number of children (*n*, %)							<0.001
0	110 (9.1)	79 (16.8)	7 (4.9)	5 (2.7)	6 (3.0)	13 (6.2)	
1	259 (21.5)	102 (21.7)	41 (28.9)	46 (25.3)	28 (13.9)	42 (19.9)	
≥2	837 (69.4)	288 (61.4)	94 (66.2)	131 (72.0)	168 (83.2)	156 (73.9)	
Number of chronic diseases (*n*, %)							<0.001
0	103 (8.5)	54 (11.5)	5 (3.5)	7 (3.8)	31 (15.3)	6 (2.8)	
1	265 (22.0)	142 (30.3)	17 (12.0)	23 (12.6)	69 (34.2)	14 (6.6)	
2	319 (26.5)	147 (31.3)	36 (25.4)	48 (26.4)	50 (24.8)	38 (18.0)	
≥3	519 (43.0)	126 (26.9)	84 (59.2)	104 (57.1)	52 (25.7)	153 (72.5)	
Number of medicines taken (*n*, %)							0.021
0	259 (21.5)	112 (23.9)	26 (18.3)	29 (15.9)	51 (25.2)	41 (19.4)	
1	430 (35.7)	168 (35.8)	39 (27.5)	69 (37.9)	79 (39.1)	75 (35.5)	
≥2	517 (42.9)	189 (40.3)	77 (54.2)	84 (46.2)	72 (35.6)	95 (45.0)	
Number of painful areas (*n*, %)							<0.001
0	637 (52.8)	300 (64.0)	68 (47.9)	46 (25.3)	128 (63.4)	95 (45.0)	
1	93 (7.7)	38 (8.1)	4 (2.8)	14 (7.7)	19 (9.4)	18 (8.5)	
≥2	476 (39.5)	131 (27.9)	70 (49.3)	122 (67.0)	55 (27.2)	98 (46.4)	
Depressive symptoms (*n*, %)							<0.001
No	889 (73.7)	391 (83.4)	99 (69.7)	93 (51.1)	170 (84.2)	136 (64.5)	
Yes	317 (26.3)	78 (16.6)	43 (30.3)	89 (48.9)	32 (15.8)	75 (35.5)	
Smoking history (*n*, %)							<0.001
Never	675 (56.0)	262 (55.9)	69 (48.6)	104 (57.1)	134 (66.3)	106 (50.2)	
Formal	389 (32.3)	123 (26.2)	61 (43.0)	74 (40.7)	48 (23.8)	83 (39.3)	
Current	142 (11.8)	84 (17.9)	12 (8.5)	4 (2.2)	20 (9.9)	22 (10.4)	
Drinking history (*n*, %)							0.116
Never	950 (78.8)	386 (82.3)	107 (75.4)	137 (75.3)	161 (79.7)	159 (75.4)	
Current	256 (21.2)	83 (17.7)	35 (24.6)	45 (24.7)	41 (20.3)	52 (24.6)	
Sleep quality (*n*, %)							<0.001
Good sleep quality	471 (39.1)	235 (50.1)	51 (35.9)	57 (31.3)	68 (33.7)	60 (28.4)	
Poor sleep quality	735 (60.9)	234 (49.9)	91 (64.1)	125 (68.7)	134 (66.3)	151 (71.6)	
Napping duration (*n*, %)							<0.001
No napping	530 (43.9)	205 (43.7)	65 (45.8)	79 (43.4)	88 (43.6)	93 (44.1)	
Short nap duration	351 (29.1)	177 (37.7)	33 (23.2)	43 (23.6)	50 (24.8)	48 (22.7)	
Long nap duration	325 (26.9)	87 (18.6)	44 (31.0)	60 (33.0)	64 (31.7)	70 (33.2)	

* Data are presented as mean ± standard deviation (SD).

** P-values were obtained by analysis of variance (ANOVA), and P < 0.001.

The participants were divided into five groups according to physical frailty and cognitive function status. The differences between the participants in the groups are shown in Table 1. There were statistically significant differences in age, education, marital status, economic source, number of children, number of chronic diseases, number of medicines taken, number of painful areas, depressive symptoms, smoking history, sleep quality, and nap duration according to the results of the chi-squared test and one-way analysis of variance (ANOVA) (*P* < 0.001).

The association between sleep quality and frailty and cognitive function is shown in Table 2, which is presented before and after adjustment for potential confounders. Compared to older adults with good sleep quality in the robust group, the results showed that those with poor sleep quality had the significantly higher prevalence of cognitive frailty (OR 3.06; 95% CI 2.11–4.46; *P* < 0.001) before the adjustments. Poor sleep quality was associated with higher risk of cognitive frailty (OR 2.95; 95% CI 2.04–4.25; *P* < 0.001) after adjusting for sociodemographic factors. Finally, the estimated OR for poor sleep quality changed slightly (OR 2.53; 95% CI 1.78–3.59; *P* < 0.001) after adjusting for all the covariates compared with the robust participants with good sleep quality. In addition, in this study, sleep quality was also positively associated with physical pre-frailty, physical frailty, and cognitive impairment. More details are presented in Table 2.

**Table 2:** Associations of sleep quality with cognitive frailty according to unadjusted and adjusted logistic regression models (*n* = 1,206).

	**Robust**	**Physical pre-frailty**			**Physical frailty**			**Cognitive impairment**			**Cognitive frailty**		
		**OR**	**95% CI**	***P*-value**	**OR**	**95% CI**	***P*-value**	**OR**	**95% CI**	***P*-value**	**OR**	**95%CI**	***P*-value**
**Model** ^**a**^													
Good quality sleep	Reference	Reference			Reference			Reference			Reference		
Poor sleep quality	Reference	2.03	1.34–3.07	0.001	2.92	1.91–4.45	<0.001	2.11	1.46–3.05	<0.001	3.06	2.11–4.46	<0.001
**Model** ^**b**^													
Good quality sleep	Reference	Reference			Reference			Reference			Reference		
Poor sleep quality	Reference	1.94	1.30–2.91	0.001	2.90	1.93–4.35	<0.001	1.99	1.41–2.82	<0.001	2.95	2.04–4.25	<0.001
**Model** ^**c**^													
Good quality sleep	Reference	Reference			Reference			Reference			Reference		
Poor sleep quality	Reference	1.79	1.22–2.64	0.003	2.20	1.53–3.16	<0.001	1.98	1.40–2.79	<0.001	2.53	1.78–3.59	<0.001

Model^a^: unadjusted.

Model^b^: adjusted for age, sex, ethnicity, residence, education, marital status, economic source, income, and number of children.

Model^c^: adjusted for age, sex, ethnicity, residence, education, marital status, economic source, income, number of children, number of chronic diseases, number of medicines taken, number of painful areas, depressive symptoms, smoking history, and drinking history.

Reference: The robust group composed of older adults with good sleep quality was the reference group.

OR, odds ratio; CI, confidence interval.

Table 3 shows the association between nap duration and cognitive frailty. This study used older adults who did not take naps in the robust group as a reference, and the results showed that short nap duration was associated with low prevalence of cognitive frailty (OR 0.57; 95% CI 0.36–0.90; *P* = 0.016) before adjusting, but long nap duration was significantly associated with high risk of cognitive frailty among the older adults (OR 2.04; 95% CI 1.28–3.24; *P* = 0.003) at the same time. In addition, after adjusting for age, sex, ethnicity, residence, education, marital status, economic source, income, number of children, short nap duration decreased the risk of cognitive frailty (OR 0.56; 95% CI 0.36–0.88; *P* = 0.012), but decreased the risk of cognitive frailty, but long nap duration increased the risk of cognitive frailty (OR 2.08; 95% CI 1.32–3.27; *P* = 0.001). Finally, short nap duration was significantly associated with lower risk of cognitive frailty (OR 0.60; 95% CI 0.40–0.89; *P* = 0.012) after adjusting for all the covariates. Meanwhile, long nap duration in the older adults was associated with higher prevalence of cognitive frailty (OR 1.77; 95% CI 1.19–2.64; *P* = 0.003). Nap duration was associated with physical pre-frailty, physical frailty, and cognitive impairment. More details on these associations are presented in Table 3.

**Table 3:** Associations of nap duration with cognitive frailty according to unadjusted and adjusted logistic regression models (*n* = 1,206).

	**Robust**	**Physical pre-frailty**			**Physical frailty**			**Cognitive impairment**			**Cognitive frailty**		
		**OR**	**95% CI**	***P*-value**	**OR**	**95% CI**	***P*-value**	**OR**	**95% CI**	***P*-value**	**OR**	**95% CI**	***P*-value**
**Model^**a**^**													
No napping	Reference	Reference			Reference			Reference			Reference		
Short nap duration	Reference	0.51	0.31–0.85	0.009	0.56	0.34–0.92	0.022	0.61	0.40–0.94	0.025	0.57	0.36–0.90	0.016
Long nap duration	Reference	1.70	1.03–2.81	0.039	1.91	1.16–3.15	0.011	1.67	1.08–2.58	0.022	2.04	1.28–3.24	0.003
**Model** ^**b**^													
No napping	Reference	Reference			Reference			Reference			Reference		
Short nap duration	Reference	0.53	0.32–0.86	0.011	0.57	0.35–0.91	0.019	0.65	0.43–0.97	0.035	0.56	0.36–0.88	0.012
Long nap duration	Reference	1.63	1.00–2.66	0.049	1.87	1.16–3.01	0.010	1.78	1.17–2.70	0.007	2.08	1.32–3.27	0.001
**Model** ^**c**^													
No napping	Reference	Reference			Reference			Reference			Reference		
Short nap duration	Reference	0.59	0.37–0.94	0.025	0.63	0.41–0.96	0.032	0.66	0.44–0.98	0.041	0.60	0.40–0.89	0.012
Long nap duration	Reference	1.60	1.01–2.52	0.045	1.80	1.18–2.72	0.006	1.71	1.14–2.58	0.010	1.77	1.19–2.64	0.003

Model^a^: unadjusted.

Model^b^: adjusted for age, sex, ethnicity, residence, education, marital status, economic source, income, and number of children.

Model^c^: adjusted for age, sex, ethnicity, residence, education, marital status, economic source, income, number of children, number of chronic disease, number of medicines taken, number of painful areas, depressive symptoms, smoking history, and drinking history.

Reference: The robust group composed of older adults who did not take naps was the reference group.

OR, odds ratio; CI, confidence interval.

## Discussion

This study is the first to investigate the association between sleep quality and nap duration, and cognitive frailty among older adults in nursing homes. Our study showed that older adults with poor sleep quality and long nap duration were at the higher risk of cognitive frailty in nursing homes than robust older adults. However, short nap duration was associated with lower risk of cognitive frailty.

In our study, the prevalence of cognitive frailty among older adults in Chinese nursing homes was 17.5%. Wang et al. found that the prevalence of cognitive frailty was 15.6% among 268 older adults in nursing homes in Nanchang province (38). A possible reason for the difference in the prevalence of cognitive frailty may be related to the multiple measurements used in their study to assess cognitive impairment. However, Zhou et al. found that cognitive frailty was as high as 27.1% among 303 older adults in nursing homes in Chongqing province (7). This may be related to the lack of uniformity in the operational definitions and measurements of cognitive frailty and fewer participants in their study, resulting in the different prevalence of cognitive frailty among older adults. These studies showed high prevalence of cognitive frailty among older adults, although the results were different. At the same time, cognitive frailty has been proven to be reversible (26), and early prevention and intervention are necessary to promote healthy aging.

Notably, older adults with poor sleep quality were more likely to experience cognitive frailty in our study. Many previous studies have already investigated sleep quality from the two aspects of physical frailty and cognitive impairment, which laid the foundation for exploring the association between sleep quality and cognitive frailty. A study on community-dwelling older adults from Hong Kong found that insomnia symptoms assessed with PSQI were common among physically frail older adults (39). In addition, using the same scale to assess sleep quality, a German study including individuals aged 50–80 years found that those with poor sleep quality, difficulties initiating sleep, and short sleep time in bed were at increased risk of mild cognitive impairment (40). Poor sleep quality significantly mediates the association between frailty severity and cognitive outcomes (41). In addition, an important study investigated the association between nighttime sleep duration and cognitive frailty, and the results showed that long sleep duration (≥9 h) was significantly associated with high prevalence of cognitive frailty among community-dwelling Chinese aged 50 and older (25). It is impossible to effectively assess the sleep quality of individuals because nighttime sleep duration is only one dimension. However, our study used the PSQI, which includes multiple dimensions of sleep quality assessment, to provide a comprehensive assessment of individual sleep quality and to better investigate the association between sleep quality and cognitive frailty. Poor sleep quality is not only related to severe frailty in older adults but is also associated with cognitive impairment; thus, to a certain extent, it is associated with cognitive frailty.

Moreover, lower odds risk of cognitive frailty was observed in older adults with short nap durations, but long nap duration was associated with higher risk of cognitive frailty. Many previous studies have investigated nap duration related to both physical frailty and cognitive impairment. Most studies have supported that napping is significantly associated with quality of sleep at night. A study on self-reported napping and nocturnal sleep demonstrated that the longer the older adults napped, the worse the quality of sleep at night (42). A study on older adults living in Italian communities found that napping was associated with both personality characteristics and consequences of their nighttime sleep disturbances (43). Among Chinese older adults, Lin found that napping was associated with delayed sleep onset, shorter sleep duration, reduction in sleep duration and light sleep (44). Thus, decreased sleep quality caused by long nap duration can lead to frailty (45). Moreover, older adults with longer nap durations in American communities have been associated with worse performance on cognitive tests (46). Longer nap duration seems to be related to different degrees of frailty and cognitive impairment in older adults and is associated with higher risk of cognitive frailty. On the other hand, Japanese community-dwelling older adults took a short nap less (than 30 min) to reduce the risk of cognitive impairment (23). In contrast, older adults in China who took a short nap were more likely to have lower cognitive scores (47). Possible reasons for the differences in results may be the different measurement used to assess cognitive impairment, the groups divided by the different duration of naps and different cultural background. Given the current studies, the field of short nap duration, whether it is associated with low prevalence of frailty and cognitive frailty among older adults, still needs further studies.

Regarding the association between frailty and cognitive impairment, a review suggested that their pathophysiological mechanisms may overlap (48). However, the mechanisms underlying the association between sleep status and cognitive frailty among older adults require further studies. Studies have shown several potential mechanisms for the association between sleep status, frailty, and cognitive impairment. In terms of sleep quality, a meta-analysis of sleep and frailty showed that sleep disorders lead to high levels of inflammatory markers such as C-reactive protein (CRP) and factor VIII, and these immunological changes are one of the main causes of frailty (45). Meanwhile, a study showed that people with cognitive impairment had higher levels of CRP (49). In terms of nap duration, a study reported that excessive daytime naps and Alzheimer's dementia may have a bidirectional relationship or share common pathophysiological mechanisms (24). In addition, longer nap duration generally resulted in more sleep inertia (50), which resulted in decreased ability to think and perform (51). Meanwhile, longer nap duration can lead to lower quality sleep at night (52), which further exacerbates frailty and cognitive impairment. In addition, short nap durations may be associated with better cognitive function, which may affect cognitive frailty (23). However, the potential mechanism between short nap duration and frailty, and cognitive frailty needs to be further explored in the future.

It can be suggested that older adults in nursing homes are a group of individuals with a huge potential to develop cognitive frailty, especially those with poor sleep quality or long nap duration. As the development of cognitive frailty is reversible (26), effective interventions for older adults are necessary. National departments need to establish relevant laws and policies, improve the healthcare system, and develop a comprehensive and affordable health and social care delivery system to provide older adults in nursing homes with medical services that can intervene with sleep status and alleviate or prevent cognitive frailty.

This study has several limitations. First, because this was a cross-sectional study, it only explored the effects of sleep quality and nap duration on cognitive frailty among older adults in nursing homes, and a causal relationship between sleep quality, nap duration, and cognitive frailty could not be concluded. Second, this study used a self-report questionnaire to investigate older adults, and the information obtained had a certain measurement deviation from objective reality. Third, the participants in our study were older adults from nursing homes in Hunan province; therefore the results may not be applicable to older adults in nursing homes located in other provinces of China.

## Conclusions

In summary, poor sleep quality and long nap duration were significantly associated with high prevalence of cognitive frailty among older adults in Chinese nursing homes. However, short nap duration decreased the risk of cognitive frailty. As nursing homes can manage older adults in a unified way, taking reasonable intervention measures, including sleep quality and nap duration, can provide new ways to effectively prevent cognitive frailty in older adults in nursing homes.

## Data availability statement

The original contributions presented in the study are included in the article, further inquiries can be directed to the corresponding author by email.

## Ethics statement

The study involving human participants were reviewed and approved by the Institutional Review Board of Xiangya School of Public Health publicly. The participants provided their written informed consent to participate in this study.

## Author contributions

SLiu, ZH, YG, FZ, and HX conceived and designed the study. SLiu and SLi analyzed the data. SLiu drafted the manuscript. All the authors were involved in data collection and writing and revision of the study. All authors contributed to the article and approved the submitted version.

## Conflict of interest

The authors declare that the research was conducted in the absence of any commercial or financial relationships that could be construed as a potential conflict of interest.

## Publisher's note

All claims expressed in this article are solely those of the authors and do not necessarily represent those of their affiliated organizations, or those of the publisher, the editors and the reviewers. Any product that may be evaluated in this article, or claim that may be made by its manufacturer, is not guaranteed or endorsed by the publisher.

## References

[1] Su Z Hu Z Peng X. The impact of changes in China's family patterns on family pension functions. Int J Health Plann Manage. (2017) 32:351–62. 10.1002/hpm.2436 28736874

[2] Chu LW Chi I. Nursing homes in China. J Am Med Dir Assoc. (2008) 9:237–43. 10.1016/j.jamda.2008.01.008 18457798

[3] PanzaFSolfrizziVFrisardiVMaggiSSancarloDAddanteF. Different models of frailty in predementia and dementia syndromes. J Nutr Health Aging. (2011) 15:711–9. 10.1007/s12603-011-0126-121968870

[4] KelaiditiECesariMCanevelliM.Abellan van KanGOussetPJGillette-GuyonnetS. Cognitive frailty: rational and definition from an (IANA/IAGG) International Consensus Group. J Nutr Health Aging. (2013) 17:726–34. 10.1007/s12603-013-0367-224154642

[5] Ruan Q Yu Z Chen M Bao Z Li J He W. Cognitive frailty, a novel target for the prevention of elderly dependency. Ageing Res Rev. (2015) 20:1–10. 10.1016/j.arr.2014.12.004 25555677

[6] Qiu Y Li G Wang X Zheng L Wang C Wang C. Prevalence of cognitive frailty among community-dwelling older adults: a systematic review and meta-analysis. Int J Nurs Stud. (2022) 125:104112. 10.1016/j.ijnurstu.2021.104112 34758429

[7] Zhou Q Zhou J Ku M Wu H Xie S. The prevelence and determinants of cognitive frailty among institutionalized older adults. J Nurs Sci. (2020) 35:88–92. 10.3870/j.issn.1001-4152.2020.09.088

[8] ChyeLWeiKNyuntMSZGaoQWeeSLNgT-P. Strong relationship between malnutrition and cognitive frailty in the singapore longitudinal ageing studies (Slas-1 and Slas-2). J Prev Alzheimers Dis. (2018) 5:142–8. 10.14283/jpad.2017.4629616708

[9] KwanRYCLeungAYMYeeALauLTXu XY DaiDLK. Cognitive frailty and its association with nutrition and depression in community-dwelling older people. J Nutr Health Aging. (2019) 23:943–8. 10.1007/s12603-019-1258-y31781723

[10] Feng L Zin Nyunt MS Gao Q Feng L Yap KB Ng TP. Cognitive frailty and adverse health outcomes: findings from the Singapore longitudinal ageing studies (Slas). J Am Med Dir Assoc. (2017) 18:252–8. 10.1016/j.jamda.2016.09.015 27838339

[11] Bu Z Huang A Xue M Li Q Bai Y Xu G. Cognitive frailty as a predictor of adverse outcomes among older adults: a systematic review and meta-analysis. Brain Behav. (2021) 11:e01926. 10.1002/brb3.1926 33159430 PMC7821586

[12] Orhan FÖ Tuncel D Taş F Demirci N Özer A Karaaslan MF. Relationship between sleep quality and depression among elderly nursing home residents in Turkey. Sleep Breath. (2012) 16:1059–67. 10.1007/s11325-011-0601-2 22120045

[13] Zhu X Hu Z Nie Y Zhu T Chiwanda Kaminga A Yu Y. The prevalence of poor sleep quality and associated risk factors among Chinese elderly adults in nursing homes: a cross-sectional study. PLoS ONE. (2020) 15:e0232834. 10.1371/journal.pone.0232834 32413064 PMC7228093

[14] Fung CH Vitiello MV Alessi CA Kuchel GA Committee ANSCP Faculty. Report and Research Agenda of the American Geriatrics Society and National Institute on aging bedside-to-bench conference on sleep, circadian rhythms, and aging: new avenues for improving brain health, physical health, and functioning. J Am Geriatr Soc. (2016) 64:e238–47. 10.1111/jgs.14493 27858974 PMC5173456

[15] Crowley K. Sleep and sleep disorders in older adults. Neuropsychol Rev. (2011) 21:41–53. 10.1007/s11065-010-9154-6 21225347

[16] Zhang Z Xiao X Ma W Li J. Napping in older adults: a review of current literature. Curr Sleep Med Rep. (2020) 6:129–35. 10.1007/s40675-020-00183-x 33777656 PMC7992388

[17] Jing R Xu T Rong H Lai X Fang H. Longitudinal association between sleep duration and depressive symptoms in Chinese elderly. Nat Sci Sleep. (2020) 12:737–47. 10.2147/NSS.S269992 33117009 PMC7569072

[18] Lin L Lu C Chen W Guo VY. Daytime napping and nighttime sleep duration with incident diabetes mellitus: a cohort study in Chinese older adults. Int J Environ Res Public Health. (2021) 18:5012. 10.3390/ijerph18095012 34065152 PMC8125963

[19] Esteban-Cornejo I Cabanas-Sanchez V Higueras-Fresnillo S Ortega FB Kramer AF Rodriguez-Artalejo F. Cognitive frailty and mortality in a national cohort of older adults: the role of physical activity. Mayo Clin Proc. (2019) 94:1180–9. 10.1016/j.mayocp.2018.10.027 30871783

[20] Dominguez LJ Barbagallo M. The relevance of nutrition for the concept of cognitive frailty. Curr Opin Clin Nutr Metab Care. (2017) 20:61–8. 10.1097/MCO.0000000000000337 27749714

[21] Henry A Katsoulis M Masi S Fatemifar G Denaxas S Acosta D. The relationship between sleep duration, cognition and dementia: a mendelian randomization study. Int J Epidemiol. (2019) 48:849–60. 10.1093/ije/dyz071 31062029 PMC6659373

[22] Sun X-H Ma T Yao S Chen Z-K Xu W-D Jiang X-Y. Associations of sleep quality and sleep duration with frailty and pre-frailty in an elderly population rugao longevity and ageing study. BMC Geriatr. (2020) 20:9. 10.1186/s12877-019-1407-5 31906855 PMC6945401

[23] Kitamura K Watanabe Y Nakamura K Takano C Hayashi N Sato H. Short daytime napping reduces the risk of cognitive decline in community-dwelling older adults: a 5-year longitudinal study. BMC Geriatr. (2021) 21:474. 10.1186/s12877-021-02418-0 34454431 PMC8401113

[24] Li P Gao L Yu L Zheng X Ulsa MC Yang H-W. Daytime napping and Alzheimer's dementia: A potential bidirectional relationship. Alzheimer's Dement. (2022) 1–11. 10.1002/alz.12636 35297533 PMC9481741

[25] Zhao Y Lu Y Zhao W Wang Y Ge M Zhou L. Long sleep duration is associated with cognitive frailty among older community-dwelling adults: results from West China Health and Aging Trend Study. BMC Geriatr. (2021) 21:608. 10.1186/s12877-021-02455-9 34706663 PMC8555015

[26] Delrieu J Andrieu S Pahor M Cantet C Cesari M Ousset PJ. Neuropsychological profile of “cognitive frailty” subjects in Mapt study. J Prev Alzheimers Dis. (2016) 3:151–9. 10.14283/jpad.2016.94 27547746 PMC4991881

[27] Morley JE Malmstrom TK Miller DK. A Simple frailty questionnaire (frail) predicts outcomes in middle aged African Americans. J Nutr Health Aging. (2012) 16:601–8. 10.1007/s12603-012-0084-2 22836700 PMC4515112

[28] Dong L Qiao X Tian X Liu N Jin Y Si H. Cross-cultural adaptation and validation of the frail scale in Chinese community-dwelling older adults. J Am Med Dir Assoc. (2018) 19:12–7. 10.1016/j.jamda.2017.06.011 28757330

[29] Folstein MF Folstein SE McHugh PR. “Mini-mental state”: a practical method for grading the cognitive state of patients for the clinician. J Psychiatr Res. (1975) 12:189–98. 10.1016/0022-3956(75)90026-6 1202204

[30] Xu G Meyer JS Huang Y Du F Chowdhury M Quach M. Adapting mini-mental state examination for dementia screening among illiterate or minimally educated elderly Chinese. Int J Geriatr Psychiatry. (2003) 18:609–16. 10.1002/gps.890 12833305

[31] Li H Jia J Yang Z. Mini-mental state examination in elderly Chinese: a population-based normative study. J Alzheimers Dis. (2016) 53:487–96. 10.3233/JAD-160119 27163822

[32] Sugimoto T Sakurai T Ono R Kimura A Saji N Niida S. Epidemiological and clinical significance of cognitive frailty: a mini review. Ageing Res Rev. (2018) 44:1–7. 10.1016/j.arr.2018.03.002 29544875

[33] Buysse DJ Reynolds CF Monk TH Berman SR Kupfer DJ. The Pittsburgh sleep quality index: a new instrument for psychiatric practice and research. Psychiatry Res. (1989) 28:193–213. 10.1016/0165-1781(89)90047-4 2748771

[34] Zhang C Zhang H Zhao M Li Z Cook CE Buysse DJ. Reliability, validity, and factor structure of Pittsburgh Sleep Quality Index in community-based centenarians. Front Psychiatry. (2020) 11:573530. 10.3389/fpsyt.2020.573530 33110414 PMC7488982

[35] Fang W Li Z Wu L Cao Z Liang Y Yang H. Longer habitual afternoon napping is associated with a higher risk for impaired fasting plasma glucose and diabetes mellitus in older adults: results from the Dongfeng–Tongji Cohort of Retired Workers. Sleep Med. (2013) 14:950–4. 10.1016/j.sleep.2013.04.015 23831240

[36] Sheikh JI Yesavage JA. Geriatric Depression Scale (Gds): Recent Evidence and Development of a Shorter Version. Haworth Press (1986). p. 165–73.

[37] Dan T. Application of short form geriatric depression scale (gds-15) in Chinese Elderly. Chin J Clin Psychol. (2013) 21:402–5. 10.16128/j.cnki.1005-3611.2013.03.036

[38] Wang L Xu C Tang L Li Y Rao T. Cognitive frailty and influencing factors of the elderly in nursing institutions. Chin J Gerontol. (2021) 41:3554–7. 10.3969/j.issn.1005-9202.2021.16.045

[39] Tang JY-m Luo H Tse M Lum TY-s Wong GH-y Li SX. The relationship between insomnia symptoms and frailty in community-dwelling older persons: a path analysis. Sleep Med. (2021) 84:237–43. 10.1016/j.sleep.2021.05.039 34175659

[40] Brachem C Winkler A Tebrügge S Weimar C Erbel R Jöckel K-H. Associations between self-reported sleep characteristics and incident mild cognitive impairment: The Heinz Nixdorf Recall Cohort Study. Sci Rep. (2020) 10:6542. 10.1038/s41598-020-63511-9 32300149 PMC7162850

[41] Kaur S Banerjee N Miranda M Slugh M Sun-Suslow N McInerney KF. Sleep quality mediates the relationship between frailty and cognitive dysfunction in non-demented middle aged to older adults. Int Psychoger. (2019) 31:779–88. 10.1017/S1041610219000292 31006402

[42] Lai H-L. Self-reported napping and nocturnal sleep in Taiwanese Elderly Insomniacs. Public Health Nurs. (2005) 22:240–7. 10.1111/j.0737-1209.2005.220307.x 15982197

[43] Frisoni GB Leo DD Rozzini R Trabucchi M. Napping in the elderly and its association with night sleep and psychological status. Int Psychoger. (1996) 8:477–87. 10.1017/S1041610296002839 9116183

[44] Lin J-N. Correlates and influences of taking an afternoon nap on nocturnal sleep in Chinese elderly: a qualitative study. Geriatr Nursing. (2018) 39:543–7. 10.1016/j.gerinurse.2018.03.002 29653772

[45] Pourmotabbed A Boozari B Babaei A Asbaghi O Campbell MS Mohammadi H. Sleep and frailty risk: a systematic review and meta-analysis. Sleep Breath. (2020) 24:1187–97. 10.1007/s11325-020-02061-w 32215833

[46] Owusu JT Ramsey CM Tzuang M Kaufmann CN Parisi JM Spira AP. Napping characteristics and restricted participation in valued activities among older adults. J Gerontol A Biol Sci Med Sci. (2018) 73:367–73. 10.1093/gerona/glx166 28958012 PMC5861910

[47] Li J Cacchione PZ Hodgson N Riegel B Keenan BT Scharf MT. Afternoon napping and cognition in chinese older adults: findings from the China health and retirement longitudinal study baseline assessment. J Am Geriatr Soc. (2017) 65:373–80. 10.1111/jgs.14368 27995615 PMC6487643

[48] Fabrício D Diniz BS. Frailty and cognitive decline. Transl Res. (2020) 221:58–64. 10.1016/j.trsl.2020.01.002 32045578

[49] Mangiafico RA Sarnataro F Mangiafico M Fiore CE. Impaired cognitive performance in asymptomatic peripheral arterial disease: relation to C-reactive protein and D-dimer levels. Age Ageing. (2006) 35:60–5. 10.1093/ageing/afi219 16364935

[50] Dinges D Orne M Orne EJSR. Sleep depth and other factors associated with performance upon abrupt awakening. Sleep Res. (1985) 14:92.

[51] Milner CE Cote KA. Benefits of napping in healthy adults: impact of nap length, time of day, age, and experience with napping. J Sleep Res. (2009) 18:272–81. 10.1111/j.1365-2869.2008.00718.x 19645971

[52] Faraut B Andrillon T Vecchierini M-F Leger D. Napping: a public health issue. From epidemiological to laboratory studies. Sleep Med Rev. (2017) 35:85–100. 10.1016/j.smrv.2016.09.002 27751677

